# Evaluation of Musculoskeletal Disorders and Ergonomic Measures among French Digestive Endoscopists

**DOI:** 10.1055/a-2895-3265

**Published:** 2026-06-25

**Authors:** Clara Yzet, Jean-Baptiste Chevaux, Frederic Telliez, Mathieu Pioche, Marion Schaefer, Rodica Gincul, Stéphane Koch, Mehdi Kaassis, Julien Jézéquel, Erwan Bories, Adrien Ollier, Geoffroy Vanbiervliet, William Vanbiervliet

**Affiliations:** 1 Department of Gastroenterology 36673 Amiens-Picardy University HospitalAmiensFrance; 2 Department of Gastroenterology 26920 Nancy Regional University Hospital CenterNancyLorraineFrance; 3 Laboratoire PériTox UMR_I 01, Institut d’Ingénierie de la Santé-UFR Médecine 26993 Universite de Picardie Jules VerneAmiensHauts-de-FranceFrance; 4 Endoscopy unit 26900 Hospices Civils de LyonLyonAuvergne-Rhône-AlpesFrance; 5 Department of Gastroenterology & Hepatology 26920 Nancy University Hospital CenterNancyFrance; 6 Gastroenterology Unit 89686 Hopital Prive Jean MermozLyonAuvergne-Rhône-AlpesFrance; 7 Department of Gastroenterology 55049 Besançon University Hospital CenterBesançonBourgogne-Franche-ComtéFrance; 8 Department of Gastroenterology 55442 Cholet Hospital CentreCholetFrance; 9 Department of Gastroenterology 26990 Centre Hospitalier Universitaire de BrestBrestBrittanyFrance; 10 Gastroenterology UnitHopital Privé de ProvenceAix en ProvenceFrance; 11 Biostatistics Unit, Clinical Research and Innovation Directorate 36673 Amiens-Picardy University HospitalAmiensHauts-de-FranceFrance; 12 Department of Gastroenterology 37114 Centre Hospitalier Universitaire de Nice Hopital L'ArchetNiceFrance; 13 Physical Medicine and Rehabilitation UnitSMR Le Grand LargeMarseilleFrance

**Keywords:** Quality and logistical aspects, Preparation, Epidemiology

## Abstract

**Background** Work-related musculoskeletal disorders (WRMSDs) affect 37–89% of gastroenterologists. This study aimed to evaluate the prevalence of MSDs among French digestive endoscopists and identify associated risk factors.

**Methods** An anonymized online questionnaire about MSD was distributed to all members of the French Society of Endoscopy (SFED) over one month.

**Results** A total of 485 digestive endoscopists participated in October 2024. Most respondents were male (55%), with a median age of 48 years (IQR 37–60). About 76.3% reported experiencing work-related joint and/or musculoskeletal disorders leading to decreased efficiency (5.1%), job adaptation (3.8%), reduction of activity (2.4%), and work stoppage (2.1%). In multivariate analysis, practicing endoscopy for more than 20 years (OR 2.53 (95% CI 1.01–6.33), *p* = 0.046) and the number of hours of endoscopy practice per week were independently associated with WRMSDs (OR 1.10 (95% CI 1.04–1.18), *p* = 0.002). Performing more than 16.5 hours of endoscopy per week increased WRMSD risk (sensitivity 35%, specificity 83%). Conversely, practicing sports at least three times per week and adjusting the endoscopy schedule were associated with a lower risk.

**Conclusion** WRMSDs are frequent and impactful among French endoscopists. Targeted preventive strategies are urgently needed to protect practitioners’ health and maintain care quality.

## Introduction

Gastrointestinal endoscopy is a physically demanding activity that exposes practitioners to unique ergonomic constraints. Endoscopists are required to perform repetitive and forceful hand movements, maintain prolonged static and often awkward postures, and apply significant torque during scope manipulation. These biomechanical demands, combined with high procedural volumes and time pressure, place endoscopists at particular risk for work-related musculoskeletal disorders (WRMSDs). WRMSDs are highly prevalent among gastroenterologists, with reported rates at 62.5% (CI 52.6–71.8) across studies. 1 2 3 This wide variability likely reflects heterogeneity in study designs, definitions of WRMSDs, survey instruments, and populations studied, as well as differences in procedural volume, subspecialty practice, and ergonomic environments. The most frequently encountered conditions include carpal tunnel syndrome, De Quervain's tenosynovitis, lateral epicondylitis, and cervical pain. 1 Previous studies have identified several individual and occupational risk factors, including a high number of procedures (more than 20 per week), prolonged endoscopy time (over 16 h per week), cumulative years of practice, female sex, and engagement in complex or prolonged procedures. 1 3 4 5 6 7 In parallel with the increasing complexity and volume of endoscopic procedures, awareness of endoscopy-related WRMSDs has grown substantially. Major endoscopy societies have issued position statements, curriculum recommendations, and ergonomic guidelines aimed at preventing occupational injuries and improving practitioner well-being. 8 9 These recommendations emphasize, among other measures, the use of adjustable equipment adapted to individual body characteristics (monitor height and position, bed height), optimization of room layout, and integration of ergonomic principles into training. Although awareness is increasing, the adoption of ergonomic solutions in gastroenterology remains inconsistent. Despite their documented effects on occupational health and performance, environmental factors are still often overlooked in ergonomic assessments, and comprehensive strategies to address them remain insufficiently developed.

The objective of our study was to assess the current prevalence of WRMSDs among French digestive endoscopists, identify associated risk factors, and evaluate the rate of adherence to ergonomic recommendations.

## Methods

### Design

A computerized, anonymized questionnaire was e-mailed twice to all members of the French Society of Endoscopy (SFED) over a one-month period in October 2024. Demographic data were collected, as well as the level of endoscopic activity performed and the number and volume of endoscopies performed per week.

WRMSDs were defined as pain associated with endoscopic practice; the level of pain was also quantified (Supplementary material 1). Occupational stress (OS) among gastroenterologists was assessed using a structured, closed-ended question specifically designed to capture the presence and perceived intensity of work-related stress and based on the Nordic questionnaire. 10

The level of expertise in endoscopy was defined as 1 for the practice of upper GI endoscopy and colonoscopy, 2 if the gastroenterologist performs some simple therapeutic procedures (balloon dilation, endoscopic mucosal resection (EMR), esophagus/colon stenting), and 3 if he or she practices endoscopic retrograde cholangiopancreatography (ERCP), interventional endoscopic ultrasound (EUS), or endoscopic submucosal dissection (ESD).

### Outcomes

The main outcome was the evaluation of the WRMSDs in the past 12 months among French gastroenterologists. WRMSDs were defined as any musculoskeletal pain associated with endoscopic practice, regardless of pain intensity threshold or persistence criteria.

The secondary outcomes were:

- the prevalence of WRMSDs by anatomic sites,
- the impact of WRMSDs on work,
- the application of current ergonomic guidelines in France, and
- the factors associated with WRMSD.

### Statistical Analysis

Quantitative variables were expressed as median (interquartile range [IQR]), and the qualitative variables were expressed as percentage. A logistic regression model was used to identify factors associated with WRMSD and OS expressed as Odds Ratio (OR) (95% confidence interval [CI]). All variables identified with a *p* < 0.20 in the univariate analysis were included in a multivariable logistic model. Discriminative ability was assessed with the area under the Receiver Operating Characteristic (ROC) curve, and the optimal cut-point was determined according to the Youden index. An ordinal logistic regression with proportional odds assumption was used to identify factors associated with a higher level of stress. All tests were two-sided, and the threshold for statistical significance was set to *p* < 0.05 in the final multivariable logistic and ordinal models. Analyses were performed using R software version 4.0.3 (R Foundation for Statistical Computing, Vienna, Austria; www.r-projet.org ) through the RStudio interface Version 1.3.1093 – © 2009–2020. The protocol was approved, according to national guidelines, by the CNIL committee (Comité Consultatif sur le Traitement de l’Information en matière de Recherche dans le domaine de la Santé) and the local committee (PI2025_843_0044).

## Results

### Population

A total of 485 French digestive endoscopists, members of the SFED, responded to the online questionnaire, out of 1500 members (32.3%). The majority were men (55%), with a median age of 48 years (IQR 37–60), most of whom had been practicing endoscopy for more than 10 years ( Table 1 ). About 49% of the respondents had a level 1 in endoscopy. The median time of endoscopy was 15 hours per week with 24 endoscopy procedures per week.

**Table 1:** Characteristics of the population.

	Overall *N* = 485	With MSD *N* = 370	Without MSD *N* = 115
Men, *n* (%)	265 (55)	195 (53)	70 (61)
Age, median (IQR)	48 (37–60)	48 (38–60)	44 (36–60.5)
Body mass index, kg/m ^2^ , median (IQR)	23.2 (21.2–25.7)	23.1 (21.2–25.7)	23.4 (21.1–25.7)
Years of endoscopy practice, *n* (%) < 5 years 5 à 10 years > 10 years and < 20 years > 20 years	42 (8.6) 107 (22) 115 (23.7) 221 (45.6)	25 (6.8) 82 (22) 89 (24) 174 (47)	17 (15) 25 (22) 26 (23) 47 (41)
Type of activity, n (%) Private hospital Public/academic hospital Both	228 (47) 195 (40) 62 (13)	178 (48) 143 (39) 49 (13)	50 (43) 52 (45) 13 (11)
Level of endoscopy, n (%) 1 (upper endoscopy and colonoscopy) 2 (Dilatation) 3 (ERCP, interventional EUS, ESD)	239 (49) 120 (25) 126 (26)	180 (49) 94 (25) 96 (26)	59 (51) 26 (23) 30 (26)
Hours of endoscopy per week, median (IQR)	15 (10–18.8)	15	13
Number of procedures, week, median (IQR)	24 (15–30)	25	21

*N* = number, IQR = interquartile range, ERCP = endoscopic retrograde cholangiopancreatography, EUS = Endoscopic ultrasound, ESD = Endoscopic submucosal dissection.

### Work-related Musculoskeletal Disorders

**Fig. 1:**
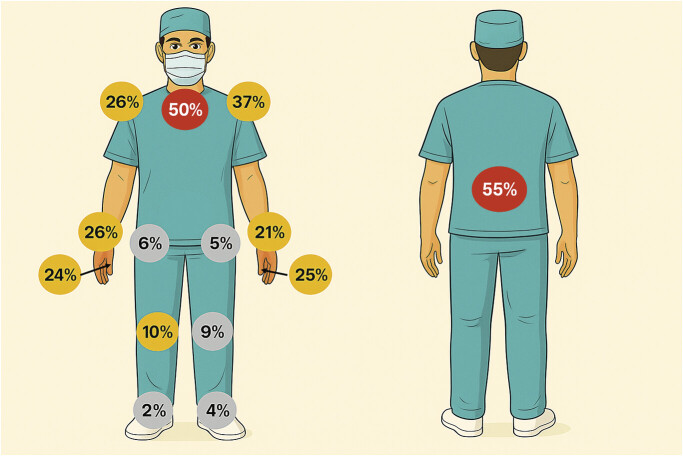
Localization and frequency of pain related to work (red bubble ≥40% of declared pain, orange 20–39%, yellow 10–19%, gray < 10%).

In all, 76.3% reported pain associated with work activity. The majority of pains were in the back (55%), neck (50%), and shoulders and hands ( Fig. 1 ). Work-related pain was specifically treated in 35% of participants. Analgesics were used by 28.4% (105/370) of the gastroenterologists suffering from WRMSD, 23.2% (86/370) had physiotherapy, and 3.5% (13/370) had surgery.

WRMSDs had an impact on work activity: 5.1% of participants reported loss of concentration, 3.8% had to adapt their activity, 2.5% had to reduce their endoscopic activity, and 2.1% reported at least one episode of work stoppage.

### Applications of Current Ergonomics Guidelines

**Fig. 2:**
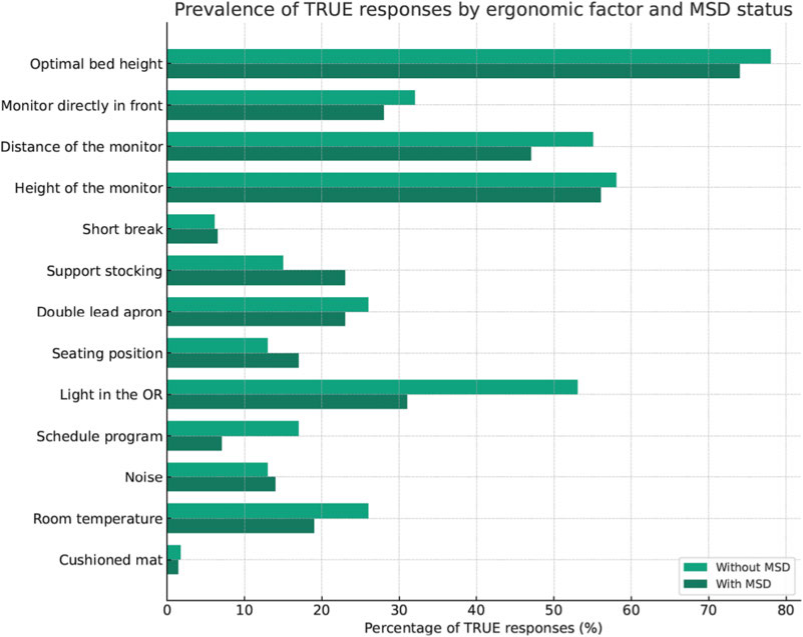
Percentage of application of ergonomic guidelines among French gastroenterologists.

Only 6.4% had received dedicated ergonomic training for preventing WRMSDs. We explored the application of ergonomic guidelines among French endoscopists. About 29.1% reported using an independent monitor, and half of the endoscopists adapted the monitor height and distance (respectively 56.1% and 49.1%) ( Fig. 2 ). Bed height was adapted in 75.2% of cases and 0.6% used a cushioned mat during their endoscopy practice. Stretching was performed by 11.7% of gastroenterologists during endoscopic procedures. Endoscopists adjusted the light intensity in 36.1% of cases, and 15.7% worked in a sitting position.

### Factors Associated with Musculoskeletal Disorders

In multivariate analysis, practicing endoscopy for more than 20 years (OR 2.53 (95% CI 1.01–6.33), *p* = 0.046) and the number of hours of endoscopy practice per week were independently associated with WRMSDs (OR 1.10 (IC 95% CI 1.04–1.18), *p* = 0.002) ( Table 2 ). Using an ROC curve analysis, 16.5 h of endoscopy per week (sensitivity: 35%, specificity: 83%) discriminated the presence of WRMSDs. The adaptation of the work environment was associated with a decreased risk of WRMSD, especially the adjustment of lighting (OR 0.21 (IC _95_ % CI 0.11–0.41), *p* < 0.001), the appropriate allocation of operative time (OR 0.22 (IC95% CI 0.09–0.55), *p* = 0.001) and the use of an endoscope support arm (OR 0.05 (IC95% CI 0–0.75), *p* = 0.042). Finally, undertaking sports activities at least 3 times per week was associated with a decreased risk of WRMSDs (OR 0.45 (IC 95% CI 0.25–0.78)) _(_ Table 2 ).

**Table 2:** Factors associated with musculoskeletal disorders: uni and multivariate analysis.

	Univariable				Multivariable		
Characteristics	*N*	OR ^1^	95% CI *^1^*	*p* -Value	OR ^1^	95% CI *^1^*	*p* -Value
Sex	485						
Men		—	—		—	—	
Women		1.40	0.91, 2.15	0.13	1.08	0.60, 1.94	0.79
Age	484	1.00	0.99, 1.02	0.57			
Body mass index (kg/m ^2^ )	484	0.99	0.94, 1.05	0.76			
Type of activity	485						
Public		—	—				
Private		1.29	0.83, 2.03	0.26			
Both		1.37	0.70, 2.82	0.37			
Level of endoscopy	485						
1		—	—				
2		1.19	0.71, 2.03	0.53			
3		1.05	0.64, 1.75	0.85			
Numbers of hours of endoscopy per week	481	1.07	1.03, 1.11	<0.001	1.10	1.04, 1.18	**0.002**
Number of procedures per week	476	1.03	1.01, 1.04	0.006	0.99	0.96, 1.02	0.67
Years of endoscopy practice	485						
<5 years		—	—		—	—	
5–10 years		2.23	1.04, 4.79	0.039	1.86	0.70, 4.93	0.21
11–20 years		2.33	1.09, 4.96	0.028	2.64	0.99, 7.07	0.052
**More than 20 years**		2.52	1.24, 5.03	0.009	2.53	1.01, 6.33	**0.046**
Sleep disturbance	485						
No		—	—		—	—	
Yes		1.48	0.97, 2.26	0.067	1.47	0.85, 2.58	0.17
Walking more than 30 minutes per day	485						
No		—	—		—	—	
Yes		0.74	0.47, 1.13	0.16	0.67	0.38, 1.16	0.16
**Sport activities**	374						
1 to 2 per week		—	—		—	—	
3 or more per week		0.57	0.35, 0.93	0.023	0.45	0.25, 0.78	**0.005**
Stress	485						
Low to very low level		—	—		—	—	
Medium		1.71	1.03, 2.84	0.037	1.64	0.83, 3.21	0.15
High to very high		1.90	1.07, 3.41	0.029	1.66	0.77, 3.58	0.19
Adaptation of the height of the exam table	485						
No		—	—				
Yes		0.79	0.47, 1.29	0.36			
Use of double screen/adapted screen	485						
No		—	—				
Yes		0.82	0.53, 1.30	0.40			
Adaptation of the distance to the screen	485						
No		—	—		—	—	
Yes		0.74	0.49, 1.13	0.16	1.34	0.73, 2.50	0.34
Adaptation of the height of the screen	485						
No		—	—				
Yes		0.90	0.59, 1.37	0.63			
Short breaks during the procedure	485						
No		—	—				
Yes		1.07	0.47, 2.75	0.88			
Use of support stocking	485						
No		—	—		—	—	
Yes		1.75	1.01, 3.17	0.055	1.95	0.96, 4.20	0.074
Use of two-parts lead apron	485						
No		—	—				
Yes		0.85	0.53, 1.38	0.49			
Use of seat during procedures	485						
No		—	—				
Yes		1.34	0.75, 2.54	0.34			
**Adaptation of the room light**	485						
No		—	—		—	—	
Yes		0.40	0.26, 0.62	<0.001	0.21	0.11, 0.41	**<0.001**
**Allocation of operative time appropriately**	485						
No		—	—		—	—	
Yes		0.36	0.19, 0.68	0.001	0.22	0.09, 0.55	**0.001**
Control of noise	485						
No		—	—				
Yes		1.04	0.57, 1.99	0.90			
Adaptation of the room temperature	485						
No		—	—		—	—	
Yes		0.67	0.41, 1.11	0.11	1.36	0.67, 2.81	0.40
**Endoscope discharge arm**	485						
No		—	—		—	—	
Yes		0.20	0.03, 1.24	0.083	0.05	0.00, 0.75	**0.042**
Cushioned mat	485						
No		—	—				
Yes		0.77	0.16, 5.46	0.76			

^1^ OR = Odds Ratio, CI = Confidence Interval.

### Stress among Gastroenterologists

Among the study population, 77.7% reported a level of stress from medium to very high. About 15.2% are receiving psychological counseling, 55.3% have sleep disorder with 63.8% reporting a feeling tired upon waking and 49.3% report sleeping between 6 and 7 hours per night.

Female sex was associated with a higher level of stress (OR 1.43 (1.01–2.03), *p* = 0.05 in the multivariable ordinal logistic regression). Conversely, a high level of endoscopy reduced the level of stress during endoscopy (OR 0.51 (0.33–0.77), *p* = 0.01) ( Table 3 ). The presence of WRMSD tended to be associated with a higher level of stress (OR 1.49 (1.00–2.22), *p* = 0.052).

**Table 3:** Factors associated with occupational stress: uni and multivariate analysis.

	Univariable		Multivariable	
	Exp(coef)	*P-* value	Exp(coef)	*P* -value
**Sex**				
Men	–	**–**	–	**–**
Women	1.45 [1.03, 2.03]	**0.03**	1.43 [1.01, 2.03]	**0.05**
**Age**	0.99 [0.98, 1.01]	0.46		
**Years of endoscopy practice**				
<5 years	–	–		
5-10 years	0.76 [0.39, 1.49]	0.43		
11 to 20 years	0.87 [0.45, 1.7]	0.69		
More than 20 years	0.98 [0.53, 1.82]	0.94		
**Level of endoscopy**				
1	–	–		
2	0.91 [0.6, 1.38]	0.66	0.87 [0.57, 1.33]	0.53
3	0.53 [0.35, 0.8]	**<0.01**	0.51 [0.33, 0.77]	**<0.01**
**Sport**				
No	–	–		
Yes	1.21 [0.81, 1.8]	0.35		
**Musculoskeletal disorders**				
No	–	**–**	–	–
Yes	1.55 [1.04, 2.3]	**0.03**	1.49 [1, 2.22]	0.052

## Discussion

WRMSDs are highly prevalent among French digestive endoscopists and represent a major occupational health concern. In our cohort, more than 75% of respondents reported suffering from WRMSD, a prevalence that is consistent with previous studies among gastroenterologists in other countries. 1 2 3 This finding supports the external validity of our results and confirms that WRMSDs remain a widespread issue in endoscopy practice.

Our study confirms that a high procedural workload is a key risk factor for WRMSDs. In particular, a greater weekly endoscopy time and a longer duration of practice (>20 years) were independently associated with an increased risk of WRMSDs. We observed that the risk increased beyond a threshold of approximately 16–16.5 h of endoscopy per week. Similar findings have been reported in a previous study of American gastroenterologists, in which performing more than 16 h of endoscopy per week was associated with a higher prevalence of WRMSDs. 3 Rather than constituting a prescriptive recommendation, this threshold should be interpreted as a signal of increased risk, highlighting the need for awareness among practitioners and institutions when organizing endoscopy workloads and schedules.

Despite the availability of guidelines on ergonomic principles in endoscopy, 9 our findings highlight that these recommendations are poorly followed in France and can lead to a number of WRMSD cases that could be prevented. This appears to be largely due to a lack of formal training in ergonomics during medical education and fellowship. In our study, only 6.4% of French gastroenterologists reported having received ergonomic training. Similarly, although educational initiatives for GI fellows appear to be more developed in the United States, previous studies reported ergonomic training rates of only 26% to 28%, highlighting the persistent lack of structured ergonomic education during endoscopy training. 4 11 Current challenges include integrating ergonomics into endoscopy training curricula, equipping procedure rooms with appropriate tools, providing team-wide training—particularly for nurses—and designating an ergonomics leader within each team to ensure accountability and continuous implementation. Physical activity emerged in our analysis as a potential protective factor against WRMSDs. To our knowledge, this is the first study among gastroenterologists to explore physical activity in this context. However, this association should be interpreted cautiously, as residual confounding cannot be excluded. In particular, healthier individuals or those with fewer symptoms may be more likely to engage in regular exercise. Nevertheless, physical activity is known to reduce WRMSD incidence in other occupational settings by approximately 30% and to improve pain control and overall well-being, particularly in musculoskeletal and rheumatologic conditions. 12 13 Promoting regular exercise and developing dedicated physical activity programs tailored to endoscopists—such as muscle-strengthening or posture-focused interventions—may therefore be a promising preventive strategy, as has been proposed for surgeons. In this context, a recent crossover trial demonstrated that a one-minute triple stretching routine significantly reduced musculoskeletal discomfort in endoscopic assistants, suggesting that brief, targeted exercise programs could be feasibly implemented in the endoscopy suite. 14

Additionally, our study brings attention to the OS experienced by gastroenterologists. The European Agency for Safety and Health at Work (EU-OSHA) defined OS as “a state of stress that occurs when there is an imbalance between a person’s perception of the constraints imposed on them by their environment and their perception of their own resources for coping with them.” Endoscopists are exposed to emergency situations and clinical decisions that inevitably exert psychological pressure. More than 50% of our cohort reported OS, with only 15% receiving psychological counselling. Our results suggest that OS is more frequent among female gastroenterologists and less frequent among those with a high level of endoscopy practice. In our study, stress was not found to be associated with WRMSDs. However, WRMSDs appeared to be associated with the occurrence of stress, both in univariate and multivariate analyses. Taken together, these findings suggest that while individuals with WRMSDs are not necessarily more stressed than those without WRMSDs, stressed individuals in the workplace are more likely to report WRMSDs. This may indicate that the presence of WRMSDs acts as a contributing factor to stress among endoscopists. Indeed, high job demands, time pressure, lack of control over work schedules, and emotional strain can lead to sustained muscle tension and maladaptive postures, especially during repetitive or prolonged procedures, pain exacerbation, and activation of inflammatory processes. 15 This combination of chronic psychological and physical load may exacerbate biomechanical stress and increase the risk of WRMSDs. Physiological studies have shown that chronic OS can disrupt the regulation of pain and inflammation through several physiological pathways, including central nervous system activation, altered cortisol response, and increased secretion of pro-inflammatory cytokines. These mechanisms may lower pain thresholds, promote musculoskeletal complaints, and impair tendon healing. 16 17 18 Persistent stress may also amplify and prolong local inflammatory responses following tissue injury. 19 20

We acknowledge several limitations in our study. First, it is a declarative cohort study. The assessment of WRMSDs is based solely on self-reported data on gastroenterologists adherent to the French society of endoscopy, which may lead to an overestimation of symptom prevalence. The questionnaire was inspired by the Nordic questionnaire, which is a validated tool, but when adapted for endoscopic purposes, it is not a validated tool. The distinction between diagnostic and therapeutic procedures was not available, as only the level of the endoscopy performed was reported. Even if the level of endoscopy was not associated with WRMSD, the specific contribution of each type of procedure remains uncertain, with therapeutic endoscopy likely to pose a higher risk. Notably, a previous study demonstrated that longer upper and lower ESD sessions were significantly associated with WRMSDs in the lower back and shoulder areas, 15 and muscle demands are markedly greater during ESD than during diagnostic endoscopy. 21 22 In addition, a potential healthy worker effect should be considered, as endoscopists with more severe musculoskeletal disorders may have reduced their procedural workload or modified their lifestyle and physical activity habits, which may have influenced the observed associations. Furthermore, due to the cross-sectional design of the study, causal relationships and temporality cannot be established, and reverse causality cannot be excluded. Similarly, the evaluation of stress levels was also declarative and not based on a validated questionnaire, which limits the accuracy of the measurement. Nevertheless, these initial results provide a foundation for further investigation in future studies using more robust tools and will help to create national ergonomics guidelines.

Despite these limitations, our study also has several strengths. To our knowledge, this is the first study to evaluate both WRMSDs and OS among French endoscopists. We obtained responses from approximately 500 physicians (one third of the SFED members), providing a substantial sample size for analysis. Finally, assessing the available equipment in the endoscopy room and its potential association with a reduced risk of WRMSD is an original aspect of our study.

In conclusion, WRMSDs are multifactorial in nature, with both physical and psychological stressors intertwined. Ergonomic education should be emphasized early in the professional pathway. Limiting weekly endoscopy time as a strategy for gastroenterologists is an essential preventive measure against WRMSDs.

**Abbreviations**
ESD: endoscopic submucosal dissection
ESGE: European Society of Gastrointestinal Endoscopy
SFED: French Society of Digestive Endoscopy

## Supplementary Material

Ergänzendes Material Supplementary Material
