# Supplementary material for: Evaluation of Musculoskeletal Disorders and Ergonomic Measures among French Digestive Endoscopists
Source: Endosc Int Open. 2026 Jun 25;14:a28953265. doi: 10.1055/a-2895-3265 (PMC13305360; doi:10.1055/a-2895-3265)
Supplement: Supplementary file 1 — Ergänzendes Material [file 10-1055-a-2895-3265_28972978.pdf]

Assessment of Musculoskeletal Disorders in Digestive Endoscopy

Supplementary Material — Questionnaire

** Indicates a required question*

SECTION 1 — GENERAL CHARACTERISTICS

1. What is your sex? *

Select one answer only.

- ○ Female
- ○ Male

2. What is your year of birth? *

Year: ______________________________

3. What is your weight (in kg)? *

Weight (kg): ______________________________

4. What is your height (in cm)? *

Height (cm): ______________________________

5. What is your dominant hand? *

Select one answer only.

- ○ Right
- ○ Left

6. What glove size do you use? *

Select one answer only (dropdown).

- ○ 6
- ○ 6.5
- ○ 7
- ○ 7.5
- ○ 8
- ○ 8.5
- ○ 9

SECTION 2 — PROFESSIONAL ROLE & ACTIVITY

7. What is your current position? *

Select one answer only.

- ○ Resident / Trainee
- ○ Fellow / Junior attending
- ○ Senior attending

8. What is your practice setting? *

Select one answer only.

- ○ Private

- ○ Public
- ○ Mixed (public & private)

9. How do you work? *

Select one answer only.

- ○ Full time
- ○ 80%
- ○ 70%
- ○ 60%
- ○ Part time (50%)
- ○ Less than part time

SECTION 3 — VOLUME & ORGANISATION OF ACTIVITY

10. What is/are your endoscopy activity level(s) according to the SFED 2023 recommendations? *

Multiple answers possible.

- ☐ Level 1: centre performing oesophagogastroduodenoscopy, colonoscopy, diagnostic endosonography, and gastrostomy placement
- ☐ Level 2: centre performing stent placement and biliopancreatic endoscopy
- ☐ Level 3: centre performing submucosal dissection, therapeutic endosonography, bariatric & metabolic endoscopy, and innovative procedures
- ☐ Level 2 and 3 combined

11. If you perform Level 2 or 3 activity, what percentage of your overall activity does this represent?

Select one answer only.

- ○ < 10%
- ○ 10 – <25%
- ○ 25% – <50%
- ○ > 50%

12. Approximately how many procedure hours do you perform per week? *

Hours/week: ______________________________

13. Approximately how many procedures do you perform per week?

Procedures/week: ______________________________

14. How do you organise your endoscopy schedule? *

Select one answer only.

- ○ Half-day sessions
- ○ Full-day sessions

15. How long have you been practising endoscopy? *

Select one answer only.

- ○ < 5 years
- ○ 5 to 10 years
- ○ 11 to 20 years
- ○ > 20 years

16. Do you participate in on-call or emergency endoscopy duties at your institution? *

Select one answer only.

- ○ Yes
- ○ No

17. If yes, how often?

Select one answer only.

- ○ < 1 per month
- ○ 1 to 2 per month
- ○ 3 to 4 per month
- ○ More than 4 per month

SECTION 4 — PAST MEDICAL & SURGICAL HISTORY

18. Do you have any history of trauma and/or surgery? *

Multiple answers possible.

- ☐ Yes, upper limbs
- ☐ Yes, lower limbs
- ☐ Yes, spine
- ☐ None

19. Do you have any visual impairment? *

Select one answer only.

- ○ Yes → proceed to question 20
- ○ No → proceed to question 21

20. If yes, what type of visual impairment do you have?

Multiple answers possible.

- ☐ Myopia
- ☐ Hyperopia
- ☐ Astigmatism

SECTION 5 — PSYCHOLOGICAL HISTORY & SLEEP

21. Have you ever received psychological support? *

Select one answer only.

- ○ Yes
- ○ No

22. What is your average sleep duration per night? *

Select one answer only.

- ○ Less than 6 hours
- ○ 6 to 7 hours
- ○ 7 to 8 hours
- ○ 8 hours or more

23. Do you experience sleep disturbance symptoms at least 3 times per week, for 3 months or more? *

Sleep disturbance symptoms include: night-time awakening, difficulty falling asleep, early morning awakening, feeling unrefreshed on waking, daytime sleepiness.

Select one answer only.

- ○ Yes → proceed to question 24
- ○ No → proceed to question 25

24. If yes, what type of sleep disturbance do you experience?

Multiple answers possible.

- ☐ Night-time awakening
- ☐ Difficulty falling asleep
- ☐ Early morning awakening
- ☐ Feeling unrefreshed on waking
- ☐ Daytime sleepiness

SECTION 6 — PAIN

25. Do you have a history of adhesive capsulitis of the shoulder or algodystrophy (Complex Regional Pain Syndrome type 1)? *

Select one answer only.

- ○ Yes
- ○ No

26. Have you ever experienced joint and/or musculotendinous pain related to your work? *

Select one answer only.

- ○ Yes → proceed to question 27
- ○ No → proceed to question 33

27. If yes, do these pains appear to be directly related to your endoscopy practice?

Multiple answers possible.

- ☐ Yes
- ☐ No

28. What is/are the location(s) of the pain?

Tick all that apply for each side.

Body region	Right-sided pain	Left-sided pain
Neck	☐	☐
Shoulder	☐	☐
Wrist	☐	☐
Thumb	☐	☐
Hand & fingers (excl. thumb)	☐	☐
Lower back	☐	☐
Interscapular	☐	☐

Knee	☐	☐
Hip	☐	☐
Ankle	☐	☐

29. What is/are the level(s) of pain intensity?

Multiple answers possible.

- ☐ Mild discomfort during the procedure
- ☐ NRS ≤ 4 during the procedure
- ☐ NRS > 4 during the procedure
- ☐ Pain persisting after the procedure
- ☐ Nocturnal pain
- ☐ Pain causing loss of efficiency or concentration
- ☐ Pain persisting for more than 3 months

30. Have you received specific treatment for this pain?

Select one answer only.

- ○ Yes → proceed to question 31
- ○ No → proceed to question 33

31. If yes, what treatment(s) have you received?

Multiple answers possible.

- ☐ Analgesic medication
- ☐ Physiotherapy / rehabilitation
- ☐ Workplace adaptation
- ☐ Reduction in endoscopy activity
- ☐ Sick leave
- ☐ Surgical treatment

32. Do you feel that you were supported by your institution in this context (management, occupational health...)?

Select one answer only.

- ○ Yes
- ○ No

SECTION 7 — ERGONOMICS

33. Have you implemented any specific workplace adaptations? *

Multiple answers possible.

- ☐ Adjusting the examination table height
- ☐ Using a dual screen / remote monitor (or more)
- ☐ Adapting the distance between yourself and the screen
- ☐ Adjusting screen height
- ☐ Using an anti-fatigue floor mat (micro-instability)
- ☐ Taking regular micro-breaks (30 s to 2 min) every 20 to 40 min during the procedure
- ☐ Wearing compression stockings
- ☐ Using a two-piece lead apron

- ☐ Adapting the weekly and/or daily schedule
- ☐ Using an endoscope support arm
- ☐ Adjusting room lighting
- ☐ Controlling ambient noise and/or atmosphere
- ☐ Adjusting room temperature
- ☐ Using a chair during procedures (performing endoscopy in a seated position)
- ☐ None

34. What do you do during breaks? *

Multiple answers possible.

- ☐ Nothing specific
- ☐ Specific stretching exercises
- ☐ Breathing exercises
- ☐ Core strengthening
- ☐ Hydration between procedure lists
- ☐ Warming up fingers/hands before the procedure list
- ☐ Screen work (checking emails, administrative tasks, etc.)
- ☐ Using a smartphone

35. Have you ever received information or training on ergonomic measures in your work as an endoscopist? *

Select one answer only.

- ○ Yes → proceed to question 36
- ○ No → proceed to question 37

36. If yes, how were you informed?

Multiple answers possible.

- ☐ Written information sheet
- ☐ Short film / video
- ☐ Expert lecture at a congress
- ☐ Occupational health consultation
- ☐ Ergonomic checklist before performing an endoscopic procedure

SECTION 8 — HEALTH & PHYSICAL ACTIVITY

37. Do you regularly practise a SPORTS activity? *

Select one answer only.

- ○ Yes → proceed to question 38
- ○ No → proceed to question 41

38. How often do you exercise?

Select one answer only.

- ○ 1 to 2 times per month
- ○ 1 to 2 times per week
- ○ 3 or more times per week

39. How long is each exercise session?

Select one answer only.

- ○ Less than 30 minutes

- 30 minutes to 1 hour
- More than 1 hour

40. What is your perceived exercise intensity? (scale of 1 to 5, where 5 = maximum effort)

Select one answer only (dropdown).

- 1
- 2
- 3
- 4
- 5

41. Do you regularly engage in PHYSICAL ACTIVITY? (e.g., walking, housework, DIY...) *

Select one answer only.

- Yes
- No

42. Do you perform regular domestic tasks (housework, DIY, gardening...) for more than 5 hours per week? *

Select one answer only.

- Yes
- No

43. Do you walk for more than 30 minutes per day? *

Select one answer only.

- Yes
- No

44. Do you climb more than 6 flights of stairs per day on foot? *

Select one answer only.

- Yes
- No

45. How would you rate your general stress level? (outside of acute stressful events: recurrent anxiety, irritability, emotional reactivity, dissatisfaction, anger, anxiolytic addictive behaviours...) *

Select one answer only.

- Very low
- Low
- Moderate
- High
- Very high

46. Have you ever received information or training on physical means available for pain management and/or wellbeing? *

Select one answer only.

- Yes
- No

47. Have you ever undergone a postural assessment and/or preventive rehabilitation assessment? *

Select one answer only.

- Yes
- No

— End of questionnaire —
